# Hypothetical Interventions on Risk Factors for Cognitive Impairment among Chinese Older Adults: An Application of the Parametric G-Formula

**DOI:** 10.3390/ijerph17031021

**Published:** 2020-02-06

**Authors:** Zi Zhou, Lun Cai, Jian Fu, Yaofeng Han, Ya Fang

**Affiliations:** 1 State Key Laboratory of Molecular Vaccine and Molecular Diagnostics, School of Public Affairs and School of Public Health, Xiamen University, Xiamen 361102, China; zhouzi@xmu.edu.cn; 2 School of Public Health, Key Laboratory of Health Technology Assessment of Fujian Province University, Xiamen University, Xiamen 361102, China; cailun@xmu.edu.cn (L.C.); rayzzbird@sina.com (J.F.); hanyaofeng@xmu.edu.cn (Y.H.); 3 School of Public Health, Xiamen University, Xiamen 361102, China

**Keywords:** cognitive impairment, g-formula, social engagement, psychological well-being, dietary intake

## Abstract

The effects of psychosocial and dietary interventions on risk of cognitive impairment is not known. The aim of this study was to estimate the 10-year risks of cognitive impairment under hypothetical interventions of psychosocial factors and dietary intake among Chinese older adults. A sample of 7377 respondents aged 65 and over was drawn from the last four waves of the Chinese Longitudinal Healthy Longevity Survey from 2002 to 2011/2012. The parametric g-formula was used to estimate the risk of cognitive impairment under independent hypothetical interventions of social engagement, psychological well-being (PWB), dietary intake, and the joint interventions of their different combination. The observed risk of cognitive impairment was 20.08% (95% confidence interval (CI): 18.81, 21.07). The risk ratios (RR) of cognitive impairment under the hypothetical interventions on higher social engagement, eating fruits at least sometimes, eating vegetables at least sometimes, positive PWB were 0.72 (95% CI: 0.65, 0.82), 0.93 (95% CI: 0.89, 0.95), 0.98 (95% CI: 0.89, 1.00) and 0.99 (95% CI: 0.98, 0.99), respectively. The RR of joint intervention was 0.64 (95% CI: 0.58, 0.73). Hypothetical interventions on psychosocial factors such as social engagement and PWB, as well as fruits and vegetable intake, were beneficial to protect older adults from cognitive impairment.

## 1. Introduction

With the largest number of older persons in the world, the aging process of China is accelerative because of the three-decade one-child policy and longer life expectancy. Elderly people’s cognitive function declines with increasing age [1,2]. The prevalence of cognitive impairment, including mild cognitive impairment (MCI) and dementia, increases with advancing age [3]. MCI represents the transition between the state of normal cognition and dementia [3]. A meta-analysis in China reported that the pooled prevalence of MCI for Chinese older adults was 12.7%, and it was 9.6% and 14.7% respectively in eastern and western China [4]. The number of older adults living with dementia is expected to increase from 9.6 million in 2010 to more than 23.3 million by 2030 [5]. Cognitive impairment severely erodes functioning, results in memory loss, decreased exercise flexibility, unresponsiveness, and so on, which affect the elderly’s quality of life seriously and create burden and stress on families [6]. Those patients who were in a late period of cognitive impairment and did not receive sufficient therapy lost activity ability and memory, followed by death. Dementia confers a huge burden on families and society in China. The costs of dementia will increase from US$47.2 billion in 2010 to US$114.2 billion by 2030 [5]. There has been no curative therapy established since research investigating the treatment of dementia has been performed worldwide for more than 20 years [7]. There is no doubt that cognitive impairment is one of the largest social care and public health challenges facing people. Interventions to delay the onset of cognitive impairment become a public health priority.

A considerable number of prospective observational studies have explored risk factors for cognitive impairment, including socio-economic characteristics, biological factors, and life behavior factors. There are limited studies on the risk factors of cognitive impairment among old adults focused on psychosocial factors [8,9] and dietary intake [10]. A systematic review showed that older adults with poor social relationships and psychological distress had an increased risk of cognitive decline or cognitive impairment [11,12,13]. Recent studies showed that older adults with higher social engagement had a lower risk of cognitive impairment but the relationship varies across different domains of social engagement [14,15]. An increased intake of vegetables is associated with slower rates of cognitive decline and decreased dementia risk among older adults [16]. These findings make it conceivable that psychosocial factors and dietary intake may play an important role in delaying the incident cognitive impairment.

Cognitive interventions at the preclinical phases could be beneficial in delaying the onset of cognitive impairment [17,18,19]. Randomized control trials (RCTs) have offered some short-term impact of interventions that can improve cognitive function such as dance [20], exercise [21], physical activities [22], and psychological intervention [23]. RCTs for long-term effects of psychosocial and dietary factors were lacking because of some practical reasons such as cost, loss to follow up, and inoperability. Moreover, due to the selective enrolment of participants into trials, the conclusions from RCTs may not always be generalized to other studies or population [24].

Therefore, the aims of this study were to estimate the 10-year risks of cognitive impairment under independent hypothetical interventions of social engagement, psychological well-being (PWB), dietary intake, and the joint interventions of their different combination by using the parametric g-formula, and compare different intervention schemes to thus provide intervention strategies among Chinese older adults aged 65 and above, using a nationally representative cohort of the Chinese Longitudinal Healthy Longevity Survey (CLHLS).

## 2. Materials and Methods

### 2.1. Data

**Figure 1:**
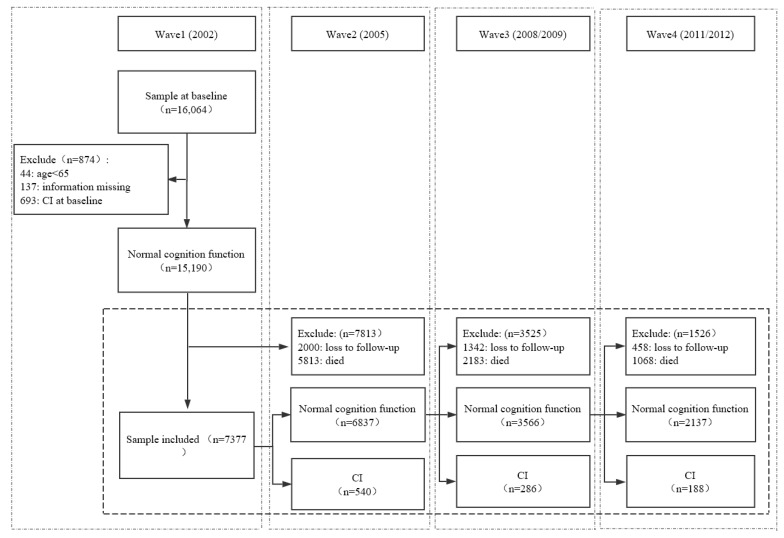
Flow chart of sample selection, CLHLS 2002–2012. * CI = cognitive impairment.

Data were drawn from a nationally representative study of community-dwelling older adults in China, the Chinese Longitudinal Healthy Longevity Survey (CLHLS). The CLHLS began in 1998 and covered counties and cities in 22 of the 31 provinces in China by using a stratified cluster sampling method, representing approximately 85% of the Chinese population. The follow-up surveys were conducted in 2000, 2002, 2005, 2008/2009, and 2011/2012. CLHLS accumulated comprehensive information of the elderly via face-to-face interviews in China, including demographics, health status and functioning, cognition testing, lifestyle and health-related behaviors, activities of daily living (ADL), and so on. Details about the study design, sampling, and data quality of the CLHLS are fully reported elsewhere [25]. The last four waves of data from 2002 to 2011/2012 were used in the current study, since 2002 (as “baseline”) was the first wave to expand the age of participants from 80 to 65 years and older. The 2002 wave of the CLHLS included 16,064 respondents aged 65 years and over. At the same time, only the respondents who completed at least one follow-up interview were included to confirm the data’s quality. Finally, a total sample of 7377 respondents aged 65 years and over was produced (Figure 1). For each participant, the follow-up was ended at the time of onset of cognitive impairment, death, loss to follow, or in the examination of wave 2011/2012, whichever occurred first.

### 2.2. Measures

#### 2.2.1. Outcome Variable

A Chinese revised version of the Mini-Mental State Examination (MMSE) was used to assess the cognitive function of participants [26]. The MMSE in CLHLS, initially modified to reflect the socioeconomic and cultural context in China, has been found to be reliable and validated [27,28,29]. Adjusting the scores of cognition function according to age and education level, the respondents were considered as cognitive impaired if their MMSE scores were below the mean scores minus 1.5 times the standard deviation [30,31].

#### 2.2.2. Intervention Variables

To be compatible with previous studies [32,33], social engagement was assessed using three categorical data on marital status (married/unmarried), living arrangement (living alone/living with others), social activity (yes/no), and two questions “Is there anyone you can talk to when you have something in mind?” (yes/no), and “Is there anyone can give you a hand when you are in trouble?” (yes/no) [25]. Each item was coded as 1 or 0. Social engagement scores ranged from 0–5; scores 0–3 were regarded as the low frequency in social engagement according to the frequency distribution, and scores 4–5 indicated the high frequency [34].

Six items were used to generate two indices, that have been documented in prior studies [35,36,37], representing the psychological well-being (PWB) among older adults, one for negative psychological well-being, and the other for positive psychological well-being. The items for negative PWB were as follows: “Do you feel the older you get the more useless you are?”, “Do you often feel lonely and isolated?”, and “Do you often feel fearful or anxious?” Five response options (always, often, sometimes, seldom, and never) were given. The three items for positive PWB were: “Are you as happy now as when you were younger?”, “Do you always look on the bright side of things?”, and “How do you think of your life at present?” Five response options (very good, good, so-so, bad, and very bad) were given for the three items. The scores ranged from 5 (very good or always) to 1 (very bad or never). Both positive PWB and negative PWB ranged from 3–15, PWB scores (−12~+12) were calculated by using positive PWB scores minus negative PWB scores [38]. The Cronbach’s alpha for the PWB was α = 0.71, which implied internal consistency. A higher score on this measure indicated a better level of PWB.

Dietary information was collected with the food frequency questionnaires. Considering the consistency of the survey waves, four common categories of food were included: vegetables, fruits, meat, and fish. The intake frequency of each food was converted to 3 categories based on the 2002 wave: very often or every day, sometimes, and seldom or never.

#### 2.2.3. Covariates

Baseline variables included age, gender, education level (illiteracy/literacy), and occupation before 65 years old (farmer/others). Age was categorized into 65–74, 75–84, 85–94, and ≥95. Our primary analysis included 6 time-varying variables: (1) residence (rural/urban); (2) exercise (yes/no); (3) smoking (never/quitted/smoking); (4) drinking (never/quitted/drinking); (5) chronic diseases (hypertension, diabetes, stroke, and cerebrovascular disease (CVD)); and (6) activity of daily living. If the respondents could not complete bathing, dressing, toileting, transferring, continence, and feeding all independently by themselves, they were regarded as disabled.

### 2.3. Hypothetical Interventions on Risk Factors for Cognitive Impairment

The parametric g-formula was used to estimate the risk of cognitive impairment under each of the following hypothetical interventions.

1. Social engagement: every participant with low social engagement was intervened on high social engagement, and all those with high social engagement were not intervened on.
2. Psychological well-being: everyone with negative PWB scores was intervened on positive scores, and the others were not intervened on.
3. Dietary intake: the respondents who seldom ate those 4 categories of food were intervened on to increase their frequency to sometimes eat those foods. The entire population was divided into two categories: every day and sometimes; (3) a: vegetables; (3) b: fruits; (3) c: meat; and (3) d: fish.
4. Interventions (1) and (2) combined.
5. Interventions (3) a and (3) b combined.
6. Interventions (3) a, (3) b, and (1) combined.
7. Interventions (3) a, (3) b, and (2) combined.
8. Interventions (3) a, (3) b, and (1)–(2) combined.

### 2.4. Statistical Analyses

Ten-years risks of cognitive impairment under the hypothetical interventions were approximately estimated by applying the parametric g-formula, which can be used to consistently estimate the effect of hypothetical intervention under the assumption that time-dependent covariates been correctly assessed at all waves.

1. Pooled regression models were used to predict the risk of cognitive impairments, each risk factor and risk of loss to follow-up or death respectively, given prior risk factor history for each 3 years period between 2002 and 2011/2012. A Kaplan–Meier estimator was used to incorporate censoring owing to death and loss to follow-up.
2. Based on these estimated models, pseudo-cohorts under each of the interventions were generated by a Monte Carlo simulation of 10,000 individuals, using the following steps:
  (a) The time-varying confounders at the next time point were simulated by the estimated regression models under the intervention of interest.
  (b) The risk of cognitive impairment and censor were simulated at the next time point and were simulated by estimated regression models in step (1) and the simulated covariates in step (2)a.
3. To predict the risk of cognitive impairment under each selected intervention, step (2) should be repeated for the entire duration of follow-up.

The population risk ratio was calculated by comparing the simulated risks under various interventions with the risk under no intervention. The 95% confidence intervals (CI) were estimated by nonparametric bootstrapping with 500 samples. Subgroup analyses were done for individuals aged ≥80 years old versus <80 years old at baseline, for male and female and for individuals with education levels of illiteracy and literacy.

Sensitivity analyses were also conducted. First, the order of the included variables was changed in the model. Second, different interaction items were tried, such as social engagement and PWB, dietary intake and social engagement and PWB, educational level and social engagement and PWB. Third, in our original research, all the covariates were included at one period back as the predictors in the parametric estimation. The analyses were conducted again using a two-period lagged model in parametric estimation. Finally, the risk ratio (RR) values were calculated when the control variables included in the model were quadratic rather than linear. All analyses were conducted using SAS 9.4 (SAS Institute, Cary, NC, USA).

## 3. Results

### 3.1. Baseline Characteristics

The baseline characteristics of the 7377 participants are depicted in Table 1, of whom 1014 participants developed cognitive impairment during the 10-year follow-up period. Over 50% of respondents were aged in the 65~ and 75~ years groups. Approximately 54% of respondents were women and over half of the respondents were illiterate (57.2%) and farmers (59.2%). The mean score of PWB was 4.39 and the proportion of high frequency of social engagement was 51.9%. The mean MMSE score was 26.11. The prevalence of cognitive impairment in participants aged 80 and over and in the western region was much higher than older adults aged less than 80 years old and in the eastern region. Meanwhile, the prevalence rate of female older adults (8.23%–11.89%) was higher than male older adults (7.41%–8.66%). Not surprisingly, illiterate elderly had a higher prevalence rate than those who were literate.

The mean of the variables of simulated data was closely related to the data under no intervention, especially in 2005 (data not shown). For example, the mean differences between the simulated and observed number of cognitive impairment prevalence rate were <0.1 during the three follow-ups.

### 3.2. Single Interventions

As presented in Table 2, the 10-year risks of cognitive impairment under 6 single hypothetical interventions were estimated by g-formula. The estimated 10-year cognitive impairment risk under no intervention was 20.08% (95% CI: 18.81, 21.07). The most effective intervention in our study was when all participants were intervened on social engagement (RR = 0.72, 95% CI: 0.65, 0.82). The other interventions were as follows: eating fruits at least sometimes: RR = 0.93, 95% CI: 0.89, 0.95; eating vegetables at least sometimes: RR = 0.98, 95% CI: 0.89, 1.00; and those with minus PWB scores were intervened on positive scores: RR = 0.97, 95% CI: 0.98, 0.99. However, hypothetical interventions of both meat and fish intake could not reduce the risk of cognitive impairment significantly.

The subgroup analyses showed evidence of a difference in independent intervention stratified by age group (see Table S1), gender (see Table S2), and education level (see Table S3). The cognitive impairment risk of young old age is lower than the risk of oldest old age. The PWB intervention has a significant effect on young old age (RR = 0.94, 95% CI = 0.93–0.98), while it has no significant effect on oldest old age, but can reduce the risk of cognitive impairment in those of oldest old age. The social engagement and fruits are protective intervention in two age groups. Men are generally less likely to develop cognitive impairment disease than women. There are no other differences between male and female under single intervention. The PWB (RR = 0.96, 95% CI = 0.93–0.98) and eating vegetables (RR = 0.98, 95% CI = 0.97–1.00) can only protect illiterate older adults.

### 3.3. Joint Interventions

Table 3 presents the results of the analyses for joint interventions after excluding two interventions, meat and fish intake, which were insignificant in the analysis of single intervention. All the combinations can reduce the cognitive impairment risk of older adults significantly. The 5th complicated intervention, which included social engagement, PWB, and vegetable and fruits intake sometimes, reduced the risk the most (RR = 0.64, 95% CI = 0.58–0.73), followed by the interventions that combined diet and social engagement (RR = 0.66, 95% CI = 0.59–0.75), social engagement combined with PWB (RR = 0.70, 95% CI = 0.64–0.80), vegetables and fruits intake at least sometimes combined with PWB (RR = 0.89, 95% CI = 0.86–0.92), and vegetable and fruit intake at least sometimes RR = 0.92, 95% CI = 0.89–0.95. Our sensitivity analysis, which tested whether the order of the variables included and the different model settings could affect the results, showed that the results were robust (sensitivity analysis results not shown).

## 4. Discussion

We estimated the cognitive impairment risk of the elderly with results under various hypothetical psychosocial and dietary intake interventions by applying the g-formula to the nationally representative cohort of CLHLS. To our knowledge, this is the first attempt to investigate interventions on cognitive impairment using the parametric g-formula among older adults. We found that social engagement, PWB, and vegetable and fruit intake were associated with decreased cognitive impairment risk, both independently and jointly. Stronger effects of PWB on cognitive impairment risk were observed among those < 80 years old compared with those ≥80 years old and among those who are illiterate compared with those who are literate.

The results from our hypothetical interventions of social engagement on cognitive impairment, which could reduce the 10-year elderly population risk of cognitive impairment by 28%, are in line with those of former studies. A recent meta-analysis concluded that older adults with multiple aspects of social relationships had a decreased risk of cognitive decline [11]. Prospective studies found that older adults with higher social engagement had a lower risk of cognitive impairment and that those with a high frequency of emotional support had better cognition function [9,34,39]. ‘Use it or lose it’ theory suggests that social engagement stimulates the brain, better social relationships may delay the onset of cognitive impairment among older adults [40]. A decrease in social engagement may lead to the disuse of the brain which in turn may accelerate the process of cognitive decline [40].

We intervened on older adults with negative PWB to bring them to a balanced condition. Our finding of significant risk reduction for cognitive impairment by PWB is consistent with the previous evidence relating PWB to cognitive impairment. The prior study also concluded that higher levels of psychological well-being were associated with better cognitive functions [41]. Several explanations could be applied to interpret the significant association between PWB and cognitive impairment. First, older adults with better PWB may be more likely to maintain healthy habits and prudent lifestyles, such as physical activity, which are important protective factors against cognitive decline. Second, it was suggested by the stress-buffering hypothesis that the association between PWB and cognitive impairment might be mediated through stress, positive well-being might buffer against stress, which in turn resulted in cognitive decline due to structural changes in the hippocampus [1].

We found that the consumption of vegetables and fruits could protect older adults from the onset of cognitive impairment and the effects of fruits were better than the effects of vegetables. There are considerable studies showing that older adults with frequent consumption of vegetables had a lower risk of cognitive impairment [16,42]. A meta-analysis concluded that the increased consumption of vegetables and fruit had positive effects on preventing cognitive impairment or dementia [43]. However, former studies found the protective effect of frequent fruit consumption appears weaker than that for vegetable [16], which was different from our study, and might be due to eating habits of the Chinese elderly. There are a variety of explanations by which the frequent vegetables and fruits intakes may be associated with decreased risk of cognitive impairment, and many of these are associated with processes that are implicated in brain aging, such as increased oxidative stress and inflammation [44].

There are several limitations in our study. First, the validity of g-formula methods relies on three common assumptions for observational study: no model misspecification, no measurement error, and no unmeasured confounding [45,46]. We have adjusted for as many potential risk factors as possible to alleviate the issue of no measurement error and no unmeasured confounding, which are inevitable in the observed study. Besides, the evidence that simulated data under no intervention from the parametric g-formula was analogous to the observed data indicated the necessary condition of the absent of model misspecification, and we conducted sensitivity analyses that showed that the results were robust across different specifications. Second, the consistency, requiring that the counterfactual outcome for all scenarios should be the same as the observed outcome under the observed exposure history, was implicitly assumed to hold in our study. This assumption may be met for social engagement and dietary intake but is less likely to meet for PWB [23]. Therefore, the hypothetical effects for PWB should be interpreted as the effect of the combination of changes that led to the decrease in PWB scores in the CLHLS population from 2002/2012. Third, caution should be taken in generalizing these results to other populations, because the risk was standardized to the distribution of confounders in the parametric g-formula in this study. Fourth, cognitive impairment was solely assessed using the MMSE, without other neuropsychological tests or clinical evaluation to verify the results. MMSE may not be sensitive to detect early stage of cognitive impairment, but this issue may be partially alleviated by setting a relatively low cut-off for MMSE in this study [47]. Finally, given the limited food categories on food frequency questionnaire in the current study, future studies should include more food categories, such as carbohydrates, sugar, and fat.

The strengths of our study included its longitudinal design and the application of the parametric g-formula with adjustment for time-dependent confounders by risk factors for cognitive impairment and simulating interventions on psychosocial factors and dietary intake.

## 5. Conclusions

Randomized trials, which are considered the ‘golden standard’, are difficult to implement. By applying parametric g-formula to longitudinal sample from the CLHLS, we found that hypothetical interventions on psychosocial factors such as social engagement and PWB, as well as fruits and vegetable intake, were beneficial to protect older adults from cognitive impairment. The g-formula is especially well suited to estimating the effects of hypothetical interventions in the presence of time-varying confounders. Our findings suggest that efforts to promote psychosocial interventions in combination with dietary intake interventions may prove to be an effective strategy for delaying the onset of cognitive impairment among older adults.

## Figures and Tables

**Table 1:** The distribution of covariates at baseline (n/%).

Variables	*N*	%	Variables	*N*	%
Cognitive functioning *	26.11 (4.6)		Drinking		
PWB *	4.39 (3.7)		not drinking	4844	65.7
Social engagement			quitting	784	10.6
low	3551	48.1	drinking	1749	23.7
high	3826	51.9	Hypertension		
Age (years)			no	6345	86.0
65~	2341	31.7	yes	1032	14.0
75~	2223	30.1	Diabetes		
85~	1794	24.3	no	7243	98.2
95~	1019	13.8	yes	134	1.8
Gender			Stroke		
male	3395	46.0	no	7087	96.1
female	3982	54.0	yes	290	3.1
Residence			Disabled		
urban	3294	44.7	no	6278	85.1
rural	4083	55.3	yes	1099	14.9
Marital status			Fruit intake		
married	4214	57.1	every day	2596	35.2
not married	3163	42.9	sometimes	2990	40.5
Living arrangements			seldom	1791	24.3
live with others	6345	86.0	Vegetable intake		
live alone	1032	14.0	every day	6587	89.3
Education			sometimes	650	8.4
illiteracy	4219	57.2	seldom	170	2.3
literacy	3158	42.8	Meat intake		
Profession			every day	2963	40.2
farmer	4358	59.1	sometimes	3160	42.8
others	3019	40.9	seldom	1254	17.0
Exercise regularly			Fish intake		
no	4517	61.2	every day	1717	23.3
yes	2860	38.8	sometimes	3621	49.1
Smoking			seldom	2039	27.6
not smoking	1594	21.6	Region		
quitting	4650	63.0	eastern	3355	45.5
smoking	1133	15.4	middle	1804	24.5
			western	2218	30.1

* mean (standard deviation); PWB = psychological well-being.

**Table 2:** Cognitive impairment risks under hypothetical single interventions.

Intervention	Risk	95% CI	RR	95% CI
Natural course (no intervention)	20.08	17.81, 21.07	1.00	——
Social engagement	14.54	12.60, 16.64	0.72	0.65, 0.82
PWB	19.46	17.51, 20.30	0.97	0.98, 0.99
Vegetables	19.73	17.63, 20.89	0.98	0.98, 1.00
Fruits	18.63	16.22, 19.94	0.93	0.89, 0.95
Meat	20.04	17.88, 21.28	1.00	0.97, 1.02
Fish	20.07	18.16, 21.22	1.00	0.97, 1.04

Notes: CI = confidence interval, RR = risk ratio, PWB = psychological well-being.

**Table 3:** Cognitive impairment risks under hypothetical joint interventions.

Intervention	Risk	95% CI	RR	95% CI
No intervention	20.08	17.81, 21.07	1.00	——
Social engagement + PWB	14.14	12.34, 16.38	0.70	0.64, 0.80
Diet (vegetables + fruits)	18.50	16.09, 19.88	0.92	0.89, 0.95
Diet (vegetables + fruits) + Social engagement	13.32	11.42, 15.46	0.66	0.59, 0.75
Diet (vegetables + fruits) + PWB	17.95	15.66, 19.15	0.89	0.86, 0.92
All factors (Social engagement + PWB + Diet (vegetables + fruits)	12.93	11.24, 15.05	0.64	0.58, 0.73

Notes: CI = confidence interval, RR = risk ratio, PWB = psychological well-being.

## Supplementary Materials

The following are available online at https://www.mdpi.com/1660-4601/17/3/1021/s1, Table S1. Cognitive impairment risks under hypothetical interventions among different age groups, Table S2. Cognitive impairment risks under hypothetical interventions among different gender groups, Table S3. Cognitive impairment risks under hypothetical interventions in different education groups.
