# Supplementary material for: Hypothetical Interventions on Risk Factors for Cognitive Impairment among Chinese Older Adults: An Application of the Parametric G-Formula
Source: Int J Environ Res Public Health. 2020 Feb 6;17(3):1021. doi: 10.3390/ijerph17031021 (PMC7036763; doi:10.3390/ijerph17031021)
Supplement: Supplementary file 1 [file ijerph-17-01021-s001.pdf]

Table S1. Cognitive impairment risks under hypothetical interventions among different age groups.

Intervention	<80 years old				≥80 years old			
	Risk	95% CI	RR	95% CI	Risk	95% CI	RR	95% CI
No intervention	16.89	14.78,18.90	1.00	——	19.55	17.00,22.77	1.00	——
Social engagement	14.01	11.74,16.57	0.83	0.72,0.93	10.28	7.49,14.14	0.53	0.38,0.76
PWB	15.94	13.84,17.96	0.94	0.93,0.98	19.49	16.62,22.51	1.00	0.98,1.02
Vegetables	16.76	14.55,18.67	0.99	0.97,1.01	19.32	16.76,22.31	0.99	0.97,1.01
Fruits	16.37	14.04,19.03	0.97	0.95,0.92	18.5	15.95,20.93	0.95	0.91,0.98
Meat	16.37	14.04,19.03	0.97	0.95,1.01	19.54	17.06,22.44	1.00	0.97,1.03
Fish	17.33	14.82,19.18	1.03	0.98,1.08	19.17	16.62,22.16	0.98	0.93,1.03

Notes: CI = confidence interval; RR = risk ratio, PWB = psychological well-being.

Table S2. Cognitive impairment risks under hypothetical interventions among different gender groups.

Intervention	Male				Female			
	Risk	95% CI	RR	95% CI	Risk	95% CI	RR	95% CI
No intervention	17.95	16.12,19.68	1.00	——	22.10	18.97,24.68	1.00	——
Social engagement	14.82	12.53,18.50	0.83	0.69,0.94	14.61	11.73,17.38	0.66	0.57,0.73
PWB	17.60	15.68,19.06	0.98	0.95,1.00	21.37	18.62,23.39	0.97	0.95,0.99
Vegetables	18.09	15.93,19.58	1.01	0.97,1.01	21.88	18.96,24.37	0.99	0.98,1.00
Fruits	17.01	14.83,19.04	0.95	0.90,0.99	20.37	17.57,22.80	0.92	0.88,0.96
Meat	18.36	16.05,20.09	1.02	0.97,1.03	21.93	19.10,24.14	0.99	0.96,1.02
Fish	17.79	15.60,19.57	0.99	0.92,1.05	22.70	19.61,25.62	1.03	0.95,1.05

Notes: CI = confidence interval, RR = risk ratio, PWB = psychological well-being.

Table S3. Cognitive impairment risks under hypothetical interventions in different education groups.

Intervention	Illiteracy				Literacy			
	Risk	95% CI	RR	95% CI	Risk	95% CI	RR	95% CI
No intervention	20.15	17.63,21.51	1.00	——	19.76	17.17,21.59	1.00	——
Social engagement	15.16	12.16,18.17	0.75	0.63,0.87	14.42	12.12,16.74	0.73	0.67,0.83
PWB	19.30	16.87,20.51	0.96	0.93,0.98	19.19	17.03,20.98	0.97	0.96,1.01
Vegetables	19.85	17.46-21.26	0.98	0.97,1.00	19.42	16.91,21.40	0.98	0.97,1.01
Fruits	18.71	16.18,20.29	0.93	0.87,0.98	18.45	15.91,20.25	0.93	0.90,0.99
Meat	20.07	17.78,21.70	1.00	0.96,1.03	19.61	16.66,21.41	0.99	0.96,1.03
Fish	20.41	17.68,18.17	1.01	0.95,1.07	19.39	16.56,21.46	0.98	0.95,1.07

Notes: CI = confidence interval, RR = risk ratio, PWB = psychological well-being.
